# Anion-regulated solvation chemistry constructs LiF-rich CEI for suppressing cathode microcracking in lithium–metal batteries

**DOI:** 10.1039/d6sc04863c

**Published:** 2026-07-16

**Authors:** Zibo Zhang, Xiaofei Liu, Yongzheng Zhang, Jian Wang

**Affiliations:** a School of Chemical Engineering, University of Science and Technology Liaoning Anshan Liaoning 114051 PR China zhangzb@ustl.edu.cn; b Helmholtz Institute Ulm (HIU) D89081 Ulm Germany; c Karlsruhe Institute of Technology (KIT) D76021 Karlsruhe Germany jian.wang@kit.edu; d Energy-Saving Building Materials Collaborative Innovation Center of Henan Province, Xinyang Normal University Xinyang P. R. China; e School of Textile and Clothing, Nantong University Nantong 226019 China

## Abstract

High-nickel layered cathodes are susceptible to interfacial side reactions, lattice oxygen release, and microcrack propagation during high-voltage cycling, leading to continuous electrolyte corrosion and rapid degradation of battery performance. To address these issues, we regulated the local solvation structure in the quasi-solid-state electrolyte to create an anion-dominated, locally high-concentration solvation environment (QSPE-AL), which promotes the preferential participation of anions in interfacial reactions and induces the formation of a LiF-rich cathode electrolyte interphase (CEI). The *in situ* polymerized polymer network provides continuous ion transport channels, which contributes to suppressing interfacial contact issues during the cycling process. The Raman spectroscopy results indicate that in QSPE-AL, more anions enter the first solvation shell around Li^+^. X-ray photoelectronic spectroscopy analysis further confirms that the LiF content in the CEI formed by QSPE-AL has increased remarkably. Owing to its high mechanical strength and interfacial stability, LiF CEI effectively buffers the volumetric stress generated during the cycling of high-nickel cathodes, inhibits the continuous penetration of electrolyte along grain boundaries, and slows the formation and propagation of microcracks in cathode particles. Electrochemical testing reveals that NCM622‖Li batteries using QSPE-AL electrolyte maintain a capacity retention rate of 88% after 300 cycles and continue to exhibit excellent stability even after more than 1000 cycles. This work provides new insights into the interface design of highly stable, high-voltage quasi-solid-state lithium–metal batteries.

## Abstract 2

An anion-dominated localized high-concentration quasi-solid-state electrolyte induces uniform LiF-rich CEI. This stable interface leads to stable battery cycling.

## Graphical abstract

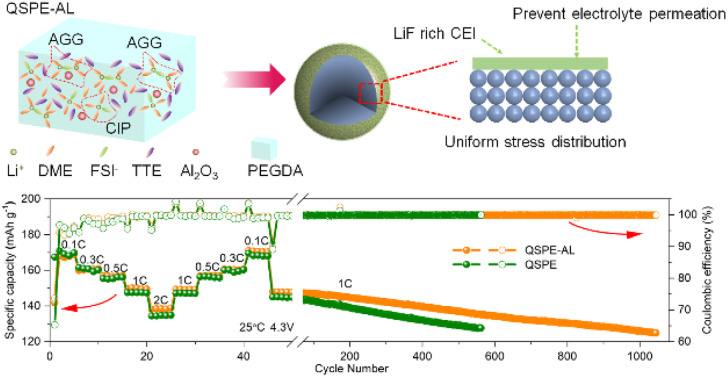

## Introduction

With the rapid development of electric vehicles, large-scale energy storage, and portable electronic devices, high-energy-density rechargeable battery systems have attracted widespread attention.^1,2^ Current commercial lithium-ion batteries are gradually approaching the theoretical energy density limit of traditional graphite anode systems.^3,4^ Therefore, the development of new energy storage systems with higher energy density has become a key research focus for next-generation electrochemical energy storage technologies.^5^ Among the many candidate systems, lithium metal is considered one of the most promising next-generation anode materials due to its theoretical specific capacity of up to 3860 mAh g^−1^ and its lowest electrochemical reduction potential (−3.040 V *vs.* SHE).^6–8^ At the same time, high-nickel layered oxide cathode materials (such as LiNi_*x*_Co_*y*_Mn_1−*x*−*y*_O_2_, NCM) offer broad application prospects in high-energy-density lithium–metal batteries due to their combination of high operating voltage and large reversible capacity.^9,10^ Among these, high-nickel cathodes such as NCM622 can achieve a reversible capacity exceeding 180 mAh g^−1^, making them a crucial component for high-energy-density lithium–metal batteries.^11^

However, high-nickel layered cathodes still face serious issues regarding interfacial and structural stability under high-voltage and long-cycle conditions. As the degree of delithiation increases, the Ni^3+^/Ni^4+^ content in the layered structure rises. This leads to a decrease in lattice oxygen stability and an increased risk of side reactions such as oxygen evolution, phase transitions, and transition metal migration.^12–14^ At the same time, high-nickel cathodes undergo significant anisotropic volume changes during repeated lithium intercalation and deintercalation cycles, causing mechanical stress to gradually accumulate within the particles.^15–17^ Microcracks form along the grain boundaries within the secondary particles when local stress surpasses its tolerance limit.^18,19^ In addition to weakening the mechanical integrity of the particles, these microcracks constantly expose fresh active surfaces, accelerating the oxidative decomposition of the electrolyte and interfacial side reactions.^20–22^ This ultimately leads to a vicious cycle of structural damage, interfacial instability, and crack propagation, which severely limits the cycle life and safety performance of high-nickel cathodes.

Recent research has demonstrated that the production of microcracks in cathode particles is causally connected to interfacial chemical stability.^23–25^ Conventional carbonate or ether-based electrolytes are prone to sustained oxidative decomposition under high-voltage conditions, leading to the formation of an organic-rich cathode electrolyte interphase (CEI) on the cathode surface.^26–29^ However, such organic CEI typically exhibit low mechanical modulus and poor chemical stability.^30,31^ They are prone to repeated fracturing and reconstruction during cycling, rendering them unable to effectively buffer the mechanical stresses generated by the volume changes of high-nickel cathodes.^32^ When the interface layer fails locally, the electrolyte continues to penetrate into the interior along grain boundaries, causing the release of lattice oxygen, dissolution of transition metals, and the accumulation of localized side reactions, all of which accelerate the initiation and propagation of microcracks.^33^ Therefore, the formation of a stable CEI that combines high mechanical strength with high chemical stability is considered a key strategy for addressing the degradation of high-nickel cathode structures.^34^ Quasi-solid polymer electrolytes (QSPEs) have attracted widespread attention for their potential to enhance battery safety and interfacial stability.^35,36^ Compared to traditional liquid electrolytes, QSPEs offer high ionic conductivity, excellent flexibility, and superior safety, effectively reducing the risk of electrolyte leakage and thermal runaway.^37–39^ However, high-voltage interface instability remains a widespread issue in traditional QSPE systems.^40^ On the one hand, these systems still contain a certain proportion of free solvent, which is prone to oxidative decomposition under high-voltage conditions. On the other hand, irregular ion transport across the interface causes a CEI with non-uniform composition and poor mechanical stability to develop on the cathode surface.^41,42^ This unstable interface fails to adapt the continuous volume fluctuations of high-nickel cathodes during cycling, making it difficult to completely suppress the propagation of microcracks inside the particles.

Recently, the localized high-concentration electrolyte (LHCE) strategy has provided a new research approach for interfacial regulation.^43,44^ By introducing an inert diluent and enhancing Li^+^–anion interactions, it is possible to effectively increase the proportion of aggregated ion pairs (AGG), reduce the activity of the free solvent, and promote the preferential participation of anions in interfacial reactions. Compared to traditional solvent-dominated interfacial reactions, anion-selective decomposition typically forms an inorganic interfacial layer rich in LiF.^45^ Extensive research has shown that LiF possesses a high mechanical modulus, excellent chemical stability, and low electronic conductivity.^46^ Therefore, a LiF-rich CEI can effectively suppress interfacial side reactions and stabilize the interface under high voltages.^47^ Furthermore, LiF-rich CEI improves the uniformity of Li^+^ transport at the interface and reduces localized concentration polarization, thereby alleviating the accumulation of mechanical stress within the particles.

Although previous studies have demonstrated that a LiF-rich interphase layer can enhance the cycling stability of high-voltage batteries, the mechanisms underlying its influence on the evolution of microcracks in high-nickel cathode particles remain poorly understood. In particular, within quasi-solid-state electrolyte systems, there is currently a lack of clear understanding regarding how the local solvation structure regulates the chemical composition of the cathode interphase (CEI), and how the LiF-rich CEI affects the internal stress distribution, grain boundary stability, and crack propagation behavior of the particles. In previous work, we achieved a synergistic enhancement of efficient Li^+^ transport in the polymer electrolyte and interface stability at both the anode and cathode by constructing a continuous and abundant anion-dominated cluster network serving as an ion transport bridge with fumed aluminum trioxide nanoparticles.^48^ While these studies primarily focused on ion transport behavior within the electrolyte and anode interface stability, there remains a lack of in-depth research on how this solvation structure regulation influences the chemical and structural evolution at the high-nickel cathode interface.

Herein, this study demonstrates an anion-dominated, locally high-concentration quasi-solid-state electrolyte system (QSPE-AL) by regulating the localized solvation structure in the quasi-solid-state electrolyte with fumed aluminum trioxide nanoparticles. Utilizing fumed aluminum trioxide nanoparticles and polymer-confined environments as new regulatory dimensions to achieve the synergistic design of dynamic solvation structure modulation and stable interface construction. Using Raman spectroscopy, XPS, EPMA, and COMSOL Multiphysics simulations, we systematically investigated the role of anions in the Li^+^ first coordination shell in promoting the formation of LiF-rich CEI. We further revealed the key role of LiF-rich CEI in buffering particle volume change stress, suppressing electrolyte penetration along grain boundaries, and mitigating the initiation and propagation of microcracks in high-nickel cathodes. This work provides new research insights and a theoretical foundation for the interface design of highly stable, high-voltage quasi-solid-state lithium–metal batteries.

## Results and discussion

**Fig. 1:**
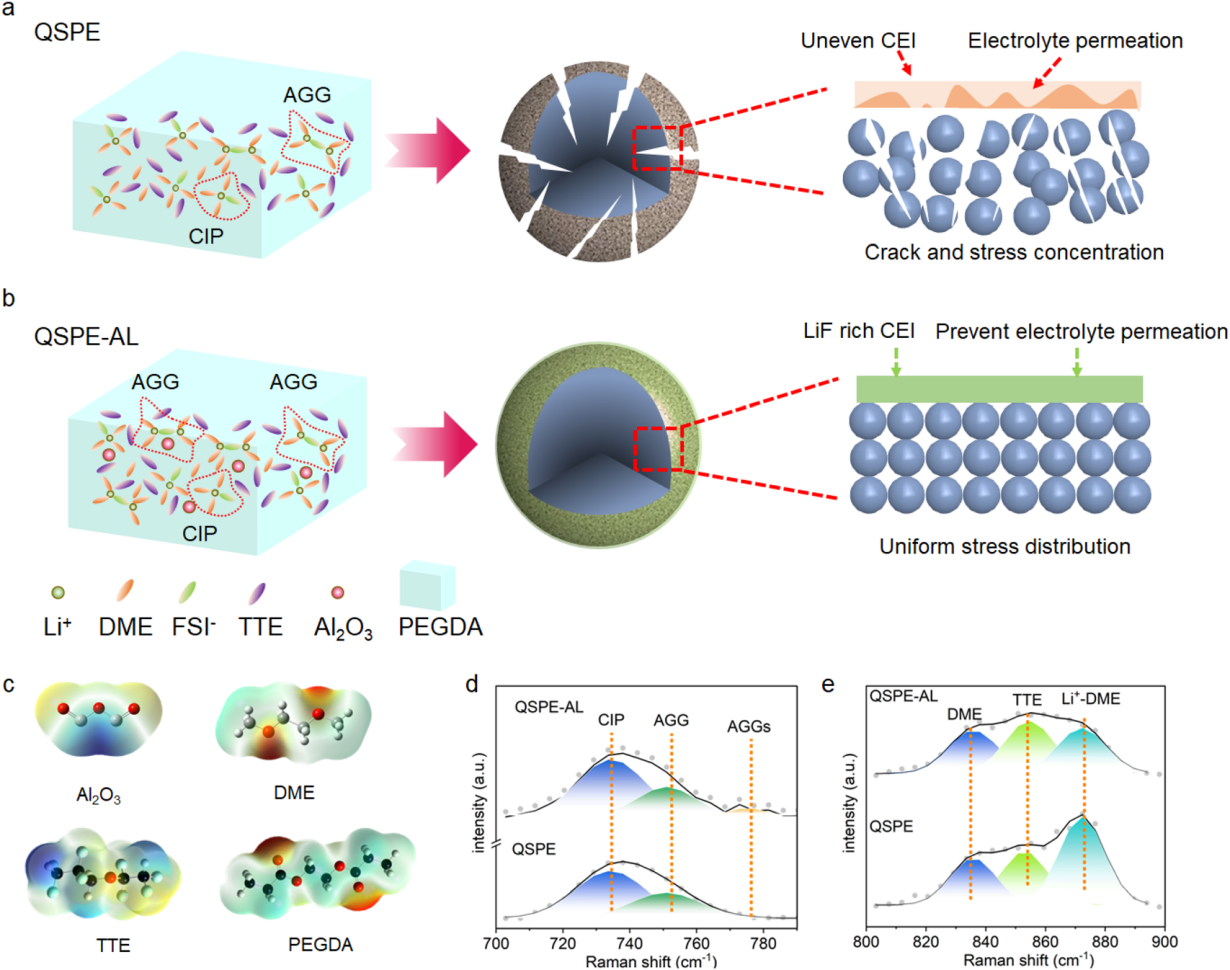
(a and b) A schematic illustration of LiF-rich CEI prevents microcrack formation in high-nickel cathodes. (c) Map of the electrostatic potential distribution for different solvents and anionic molecules (where red regions indicate electron-rich areas and blue regions indicate electron-poor areas). (d and e) Raman spectra and peak fitting results for characteristic vibrational peaks of anions and solvents in QSPE and QSPE-AL electrolytes.

Fig. 1a and b schematically illustrates the correlation mechanisms between solvation structures, interfacial chemistry, and the evolution of microcracks in cathode particles under the two electrolyte systems, QSPE and QSPE-AL. In conventional QSPE electrolytes, the Li^+^ ions are primarily surrounded by a CIP structure, with limited anion coordination and a high proportion of free solvent. This solvation environment leads to preferential oxidative decomposition of the solvent at the cathode interface, resulting in the formation of a CEI rich in organic components. Because the organic CEI has low mechanical strength and an uneven structure, it is prone to localized cracking during high-voltage cycling. As shown in Fig. 1a, for QSPE electrolyte, once the CEI layer breaks down, the electrolyte continuously penetrates into the interior of the particles along interface defects and grain boundaries, thereby triggering sustained side reactions and releasing lattice oxygen. At the same time, due to localized uneven Li^+^ transport, distinct stress concentration zones form within the particles, ultimately leading to the formation and propagation of microcracks. In contrast, in QSPE-AL, the introduction of an anion-dominated, locally highly concentrated solvated structure significantly increases the AGG coordination ratio, allowing anions to preferentially participate in interfacial reactions and form a LiF-rich inorganic CEI. The LiF-rich CEI exhibits a higher mechanical modulus and superior interfacial stability, enabling the formation of a uniform and dense protective layer. As shown in Fig. 1b, the formation of a LiF-rich CEI effectively blocks the continuous penetration of the electrolyte and uniformly distributes the volumetric stress generated by the high-nickel cathode during cycling, thereby preventing localized stress concentration. In addition, a stable CEI reduces the release of lattice oxygen and the accumulation of side reactions, further inhibiting the degradation of the particle internal structure. Consequently, QSPE-AL forms a LiF-rich CEI through its anion-dominated solvation structure. This uniform and dense CEI facilitates uniform lithium deintercalation, promotes uniform stress distribution, thereby suppressing microcrack propagation, and achieves excellent cycling stability in high-voltage lithium–metal batteries.

To elucidate the tuning mechanism of the localized solvation structure in a quasi-solid polymer electrolyte (QSPE) by fumed aluminum trioxide nanoparticles (Al_2_O_3_), this study employed electrostatic potential (ESP) analysis combined with Raman peak fitting to systematically investigate the coordination environment and ion association behavior of Li^+^ in the system. As shown in Fig. 1c, the structures associated with fumed aluminum trioxide (Al_2_O_3_), 1,2-dimethoxy-ethane (DME), 1,1,2,2-tetrafluoroethyl-2,2,3,3-tetrafluoropropylether (TTE), poly(ethylene glycol) diacrylate (PEGDA), all exhibit distinctly different charge distribution characteristics. The ether oxygen sites in the DME molecule form a region of significantly negative electrostatic potential, indicating that DME possesses strong electron-donating capabilities and can form stable coordination bonds with Li^+^. Therefore, DME is the primary Li^+^ solvating molecule in the system. The ether oxygen and carbonyl oxygen atoms in the PEGDA molecule also have high electron density, indicating that the polymer segments can participate in the local coordination of Li^+^ and provide a continuous polar channel for ion transport. In contrast, the overall electrostatic potential distribution of the TTE molecule is relatively uniform, with only weakly negative regions present near individual fluorine atoms, suggesting that it is unlikely to participate directly in Li^+^ solvation and is more likely to act as a non-coordinating diluent. The oxygen sites on the Al_2_O_3_ surface exhibit distinct electron-rich characteristics, indicating that they possess Lewis base properties. These sites can interact with Li^+^ ions, thereby influencing the configuration and stability of the Li^+^ first solvation shell. The addition of fumed Al_2_O_3_ forms a stable suspension, which interacts with solvent molecules and Li^+^ through Lewis acid–base interactions, thereby acting as an interfacial solvation regulator. These interfacial interactions disrupt the local coordination equilibrium of Li^+^, leading to a restructuring of the solvation structure. The S–N–S stretching vibration mode of the FSI^−^ anion can be observed at 700–800 cm^−1^, enabling the study of changes in the coordination state of the anion (Fig. 1d). In QSPE-AL system, the characteristic peaks at approximately 735 cm^−1^, 752 cm^−1^, and 775 cm^−1^ correspond to contact ion pairs (CIP), aggregated ion pair (AGG), and higher-order aggregate structures (AGGs), respectively. In the QSPE system, CIP peaks dominate, with a certain proportion of AGG structures, while AGG peaks are barely observable, indicating that Li^+^ exists primarily in a solvated state, with anions participating only to a limited extent in its first coordination shell. Upon the introduction of Al_2_O_3_, the AGG peak in the Raman spectrum of QSPE-AL was significantly enhanced, and new AGG characteristic peaks appeared, indicating the formation of a higher degree of anion aggregation in the system, *i.e.*, more FSI^−^ ions entered the first solvation shell of Li^+^. These results indicate that the introduction of Al_2_O_3_ significantly weakens the dominant coordinating role of solvent molecules toward Li^+^ and promotes the participation of anions in the construction of the Li^+^ local coordination environment. Analysis of the DME-related Raman vibrational peaks further confirmed the reconstruction of the Li^+^ solvation structure.

In QSPE-AL, the Li^+^-DME peak was markedly weakened (Fig. 1e and S1b), while the peak intensities of free DME and TTE were significantly enhanced, indicating that the introduction of Al_2_O_3_ weakened DME's direct coordination ability with Li^+^ and promoted a shift in the system from a solvent-dominated solvation structure to an anion-participating solvation structure. Fig. S1a shows the statistical distribution of peak area ratios for the various coordination structures of FSI^−^. For QSPE, the proportions of CIP, AGG, and AGGs were approximately 65%, 35%, and 0%, respectively, indicating that the system is still dominated by traditional contact ion pair structures. In the QSPE-AL system, the CIP fraction remains at 66%, while approximately 10% of original AGG structure has been replaced with AGGs structure. The formation of AGGs indicates the presence of higher order anionic aggregates in the system, reflecting a significant enhancement in the interaction between Li^+^ and FSI^−^. This anion-enriched local structure typically exhibits characteristics similar to those of a locally high-concentration electrolyte (LHCE), effectively reducing the activity of free solvents and enhancing anion-dominated interfacial reactions. Consequently, the incorporation of Al_2_O_3_ not only improves the structural stability of the polymer electrolyte as an inorganic filler, but also remodels the local solvation environment of Li^+^ through its surface Lewis acid and base sites. This regulatory effect significantly reduces the dependence of Li^+^ on DME, promotes the entry of FSI^−^ into the first coordination shell, and induces the formation of an anion-enriched structure dominated by AGG/AGGs. This localized high-concentration profile helps suppress side reactions involving free solvent at the electrode interface and promotes the preferential decomposition of FSI^−^ to form a stable CEI layer rich in LiF, thereby effectively enhancing the stability of the electrolyte/electrode interface as well as the cycling and rate performance of battery.

**Fig. 2:**
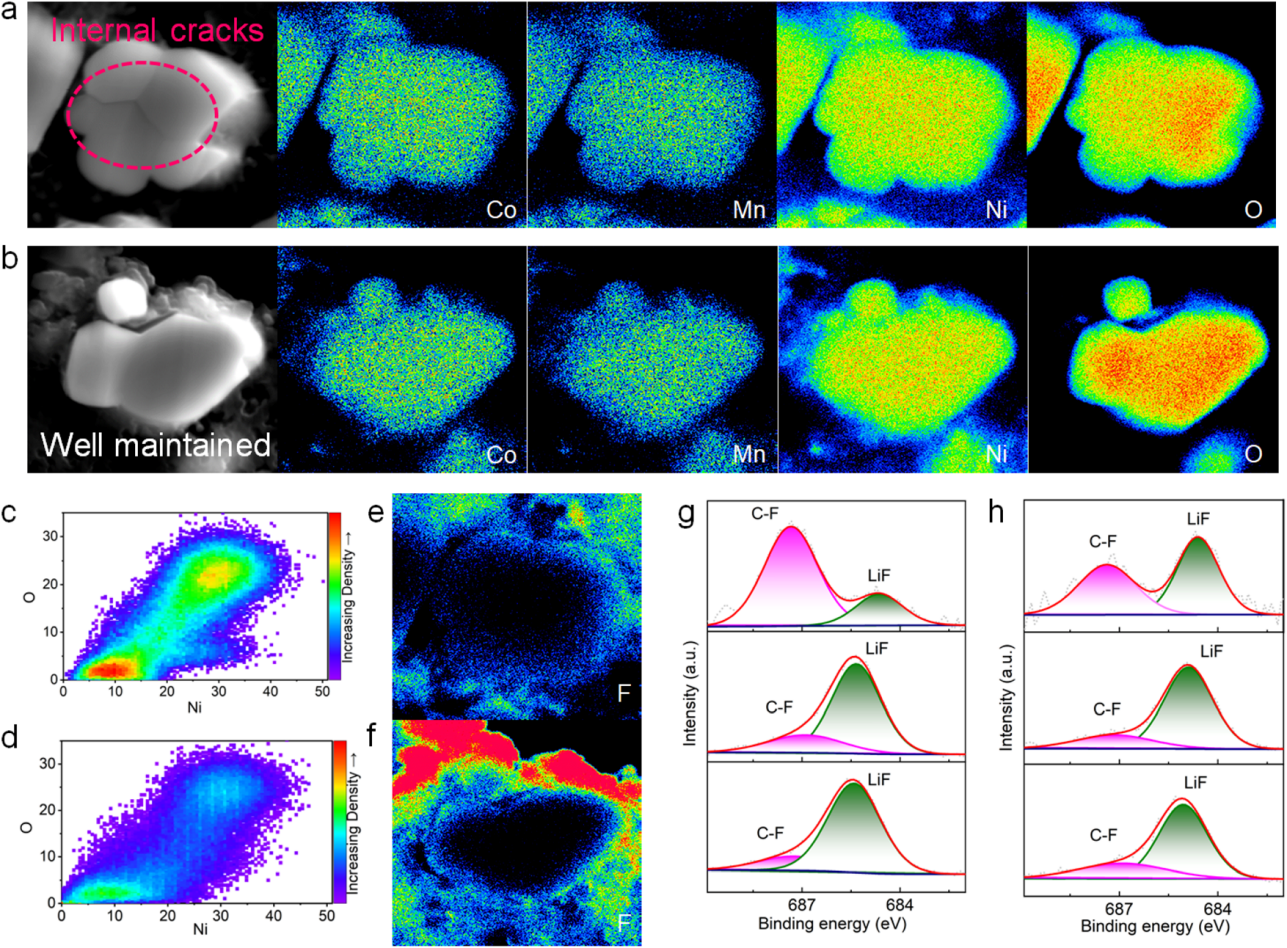
Cross-sectional electro-probe micro-analysis (EPMA) analysis of the NCM622 cathode with QSPE (a) and QSPE-AL (b). (c and d) Ni–O binary compositional distribution diagrams of NCM622 cathode with QSPE and QSPE-AL. (e and f) Distribution map of the F component in the NCM622 cathode with QSPE and QSPE-AL. (g and h) F 1s XPS spectra of NCM622 before and after etching with both electrolytes after 50 cycles.

To further elucidate the effect of LiF-rich CEI on the structural stability of the cathode, surface and interface analysis was performed on the NCM622 cathode after cycling. Fig. 2 systematically reveals the composition of the CEI under different electrolyte systems and its impact on the structural stability of high-nickel cathodes through cross-sectional EPMA elemental mapping, two-dimensional elemental correlation analysis, and XPS F 1s spectra, and further elucidates the mechanism by which LiF-rich CEI suppresses the formation of microcracks in the cathode. Fig. 2a and b show the EPMA elemental distribution results of the cathode particles in the QSPE and QSPE-AL electrolyte systems after cycling, respectively. In the conventional QSPE system (Fig. 2a), significant non-uniform elemental enrichment is observed on the particle surface, with distinct bright regions particularly visible near the particle edges. This indicates that interfacial side reactions are quite intense during the cycling process, leading to the continuous accumulation of components at the interface. At the same time, marked discontinuities in elemental distribution appear in localized regions of the particles, suggesting that the electrolyte has continuously penetrated into the interior of the particles along grain boundaries or crack regions. In particular, the QSPE electrodes exhibit non-uniform distribution in the O-element distribution map, indicating significant lattice oxygen loss and transition metal migration during the cycling process (Fig. 2a). In contrast, the elemental distribution in the QSPE-AL system (Fig. 2b) is significantly more uniform. In particular, oxygen forms a continuous and uniform distribution within the particles, with no obvious regions of elemental enrichment observed. This indicates that the LiF-rich CEI effectively prevents the continuous penetration of the electrolyte along grain boundaries, thereby maintaining the stability of the high-nickel layered structure. In the QSPE system (Fig. 2c), the Ni–O distribution exhibits distinct discretization and extends into regions with lower O content, indicating significant lattice oxygen loss and transition metal migration during cycling. This oxygen loss is typically closely associated with interfacial side reactions and structural degradation under high-voltage conditions. As lattice oxygen continues to be released, the stability of the layered structure decreases, making the interior of the particles more susceptible to localized stress concentrations and microcracks. In contrast, the Ni–O distribution in the QSPE-AL system (Fig. 2d) is significantly more concentrated, and the high-density regions are more uniform. This further indicates that the LiF-rich CEI effectively suppresses interfacial side reactions and the release of lattice oxygen, thereby maintaining the stability of the high-nickel layered structure. In QSPE (Fig. 2e), distinct regions of elemental depletion and non-uniform distribution are observed at the particle edges, indicating that after localized rupture of the interfacial film, the electrolyte continues to erode the particle surface and induce structural degradation. At the same time, a distinct high-intensity distribution appears in the boundary region, indicating the continuous accumulation of side-reaction products. In contrast, QSPE-AL exhibits a more continuous and uniform interfacial layer, indicating that the LiF-rich CEI can stably exist on the particle surface and maintain an intact interfacial structure (Fig. 2f). This stable inorganic interface effectively reduces direct contact between the electrolyte and the active particles, thereby minimizing localized side reactions and stress accumulation. Therefore, LiF-rich CEI not only enhances the chemical stability of the interface but also slows the propagation of microcracks along grain boundaries by preventing electrolyte penetration. The F 1s XPS spectra of the cathode surface after cycling under different systems, along with the peak fitting results, further illustrate the differences in surface composition. In QSPE (Fig. 2g), the F 1s spectrum consists primarily of a strong C–F peak (∼687 eV), while the LiF peak (∼685 eV) is relatively weak, indicating that the interfacial film consists mainly of organic fluorinated byproducts.^49^ Such organic CEIs have low mechanical strength and are prone to fracture during high-voltage cycling. In contrast, in the QSPE-AL electrode (Fig. 2h), the LiF peak is markedly enhanced, while the C–F peak is significantly attenuated. The proportion of LiF has increased significantly, indicating the formation of an inorganic-dominated LiF-rich CEI at the interface, which effectively buffers the mechanical stress caused by particle volume changes during cycling. At the same time, compared to QSPE (Fig. S2), a weaker Ni 2p signal (contributed by nickel-containing CEI components, such as NiF_2_) was observed (Fig. S3), indicating that the fluorine-rich CEI suppresses the dissolution of transition metal, thereby maintaining structural stability.

**Fig. 3:**
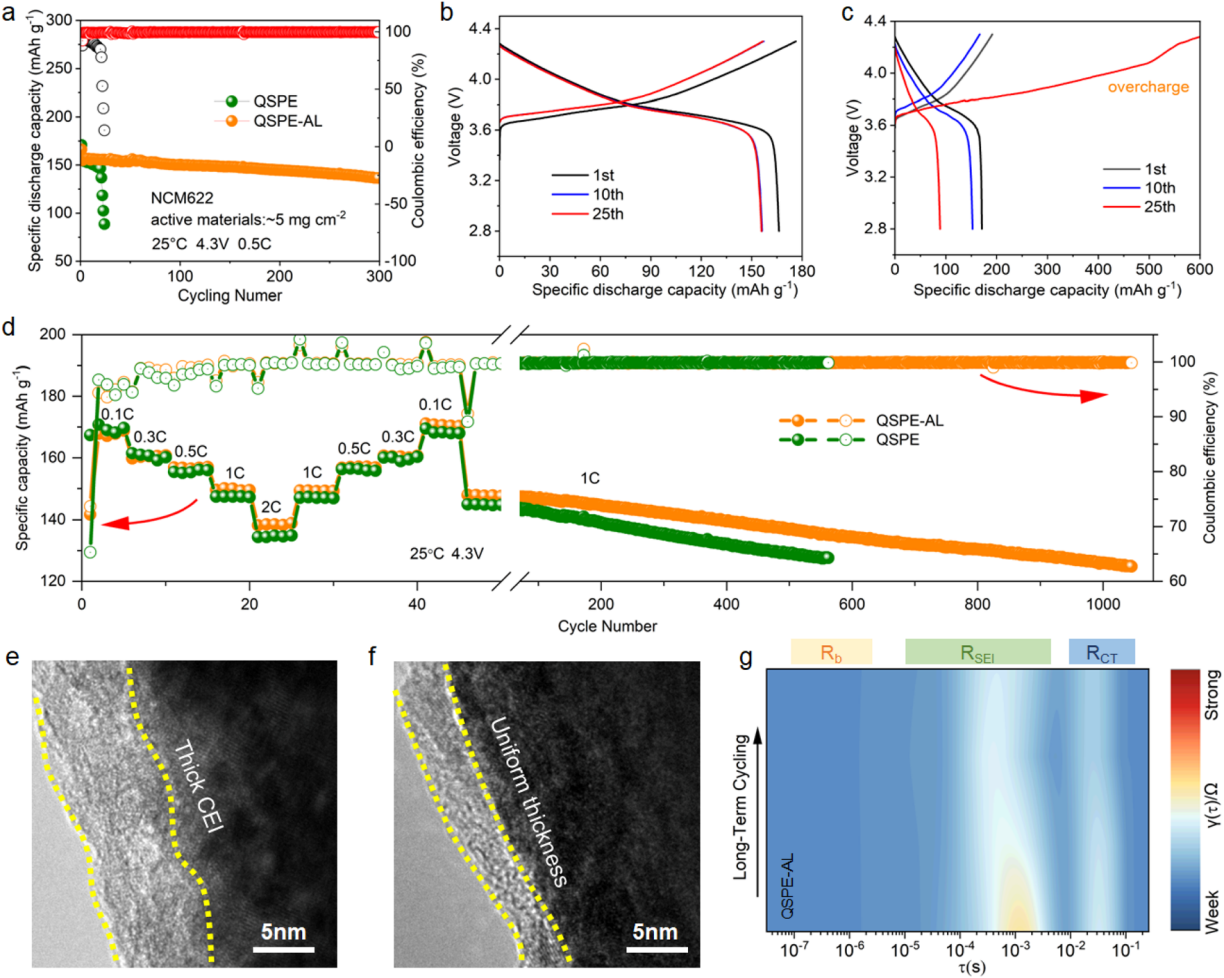
Comparison of electrochemical performance among different electrolyte systems. (a) Cycle performance and coulombic efficiency of NCM622‖Li batteries in different electrolytes. (b and c) Charge–discharge curves at different cycles with QSPE-AL and QSPE. (d) Comparison of rate performance and long cycle stability with QSPE and QSPE-AL. HRTEM images of the CEI in the different electrolytes of (e) QSPE, (f) QSPE-AL. (g) The distribution of relaxation time (DRT) analysis of EIS Nyquist plots for different cycles with QSPE-AL.

To evaluate the impact of the solvation structure on battery performance, the electrochemical behavior of NCM622‖Li batteries was further tested under different electrolyte systems. As shown in Fig. 3a, at a high loading of ∼5 mg cm^−2^, the QSPE-AL battery exhibits significantly better cycling stability than QSPE. After 300 cycles, QSPE-AL still maintains a discharge capacity of approximately 138 mAh g^−1^, with a capacity retention rate of about 88% and a coulombic efficiency that remains stable at approximately 99.7%. This polymer electrolyte ensures the formation of effective ionic pathways between the electrolyte and the electrode materials. Corresponding energy-dispersive X-ray spectroscopy (EDS) analysis indicates that the *in situ*-generated QSPE-AL exhibits good diffusion connectivity with the cathode material (Fig. S4). In contrast, during the first 30 cycles, the capacity of the QSPE battery rapidly declined from approximately 154.7 mAh g^−1^ to less than 88 mAh g^−1^, accompanied by significant fluctuations in efficiency. This difference primarily derives from variations in the stability of the interfacial layer. In QSPE, the electrolyte undergoes continuous oxidative decomposition at the interface, leading to the gradual thickening and rupture of the CEI, which ultimately induces stress concentration and microcrack propagation within the NCM622 particles. In contrast, the LiF-rich CEI formed in QSPE-AL possesses a higher mechanical modulus and greater chemical stability, effectively buffering volume changes during cycling. Fig. 3b further shows that the charge–discharge curves of the QSPE-AL battery are highly consistent at cycles 1, 10, and 25, indicating minimal growth in interfacial impedance and stable Li^+^ transport. This suggests that the LiF-rich CEI is capable of maintaining a complete and continuous ion transport pathway. In contrast, the QSPE battery exhibited severe abnormal charging behavior on the 25th cycle (Fig. 3c). Its charging capacity exceeded 550 mAh g^−1^ and continued to rise in the high-voltage region, showing clear signs of overcharging. This indicates that, due to CEI breakdown and the propagation of microcracks in the cathode, electrolyte continuously penetrated into the interior of the particles, triggering violent side reactions and parasitic oxidation processes. Rate performance testing (Fig. 3d) further demonstrates the advantages of QSPE-AL in terms of interfacial stability. Over the 0.1–2C range with a loading of 2.5 mg cm^−2^, its capacity decreased steadily from approximately 170 mAh g^−1^ to approximately 138 mAh g^−1^, whereas QSPE exhibited more pronounced capacity decay at high rates. This indicates that a stable CEI can reduce interfacial impedance and maintain rapid Li^+^ transport. The results of the long cycling tests more directly demonstrate the important role of LiF-rich CEI in suppressing microcrack propagation. QSPE-AL maintained a capacity of approximately 125 mAh g^−1^ after more than 1000 cycles, whereas QSPE had already degraded to approximately 127 mAh g^−1^ after about 550 cycles. This indicates that a stable inorganic CEI can effectively slow down the degradation of the internal particle structure and interfacial instability. As shown in Fig. 3e, following QSPE cycling, a CEI film of non-uniform thickness formed on the electrode surface, with the thickness fluctuating around 8 nm, while significant localized thickening was observed. This indicates that electrolyte decomposition reactions continued to occur during the cycling process, leading to the continuous growth of the interfacial film. In contrast, the CEI film formed by the optimized QSPE-AL system (Fig. 3f) has a thickness of 5 nm and is uniformly distributed across the electrode surface, demonstrating a denser and more stable interfacial structure. The DRT analysis results are shown in Fig. 3g and corresponding EIS spectra is shown in Fig. S5. During long-term cycling with QSPE-AL, the peak intensity corresponding to *R*_b_ remained essentially stable, indicating that the conductivity of the electrolyte itself changed only slightly. Only a slight increase was observed in the *R*_SEI_ region, demonstrating that the formed interfacial film exhibits good structural stability. No significant enhancement was observed in the *R*_CT_ region, indicating that the charge transfer kinetics at the electrode/electrolyte interface are effectively maintained over long cycling periods. Combined with the TEM results, it is evident that a uniform and thin CEI layer helps reduce interfacial ionic transport resistance, thereby improving the cyclic stability of the battery. Compared to QPSE (Fig. S6), *R*_SEI_ and *R*_CT_ were significantly enhanced. Combined with TEM results, it can be seen that side-reactions make it difficult to form a stable interface, and ion transport is also impeded. In conventional QSPE systems, the CEI formed on the surface of cathode particles consists primarily of organic components, which have a low mechanical modulus and insufficient interfacial stability. During cycling, this interfacial layer is prone to localized cracking, leading to uneven Li^+^ transport and localized stress concentration.

**Fig. 4:**
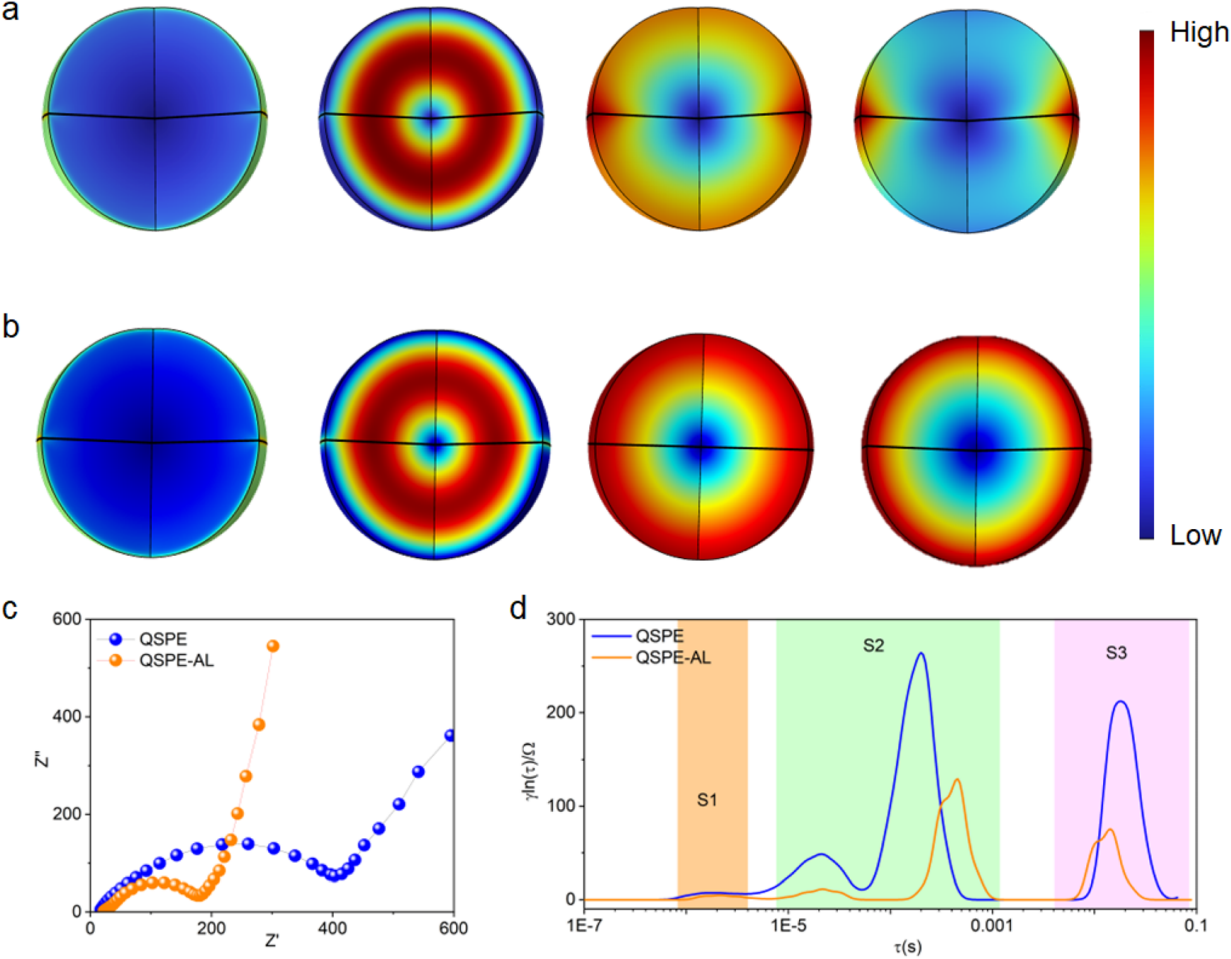
Simulated evolution of stress of NCM622 during lithiation delithiation cycles with QSPE (a) and QSPE-AL (b). (c) EIS spectra of cell with both electrolyte after 50 cycles. (d) DRT analysis of the impedance data.

To further elucidate the effects of improved interfacial reaction kinetics on the cathode, COMSOL Multiphysics simulations were performed. As shown in Fig. 4a, a distinct high-response ring appears at the particle edges as cycling progresses. This indicates that during delithiation, the Li^+^ concentration gradient increases rapidly, resulting in significant non-uniform volumetric contraction within the particle, suggesting that localized stress accumulation is beginning to occur in the interface region. As the cycles continue, the high-response region gradually expands from the particle surface toward the interior, forming a gradient distribution that propagates from the periphery toward the center. This indicates that interfacial stress has begun to be transmitted into the particle interior, which can easily induce localized structural distortions in the grain boundary regions. With repeated deep cycling, distinct localized high-stress regions appear at the edges of the particles, with significant stress concentrations forming particularly at the intersections of transverse grain boundaries. These regions are typically where microcracks in high-nickel cathodes are most likely to initiate. As cycling continues, stress accumulates along the grain boundaries, leading to the formation of through-cracks. After long-term cycling, unstable CEI leads to localized stress concentration, which causes the cathode particles to crack and eventually fragment (Fig. S7). In contrast, the QSPE-AL system exhibits significantly more uniform and stable local distribution characteristics. Although a certain concentration gradient still exists during the delithiation process, the distribution of high-response regions is more uniform, and no distinct localized sharp stress bands are observed (Fig. 4b). This indicates that LiF-rich CEI is capable of maintaining a stable and continuous Li^+^ transport pathway, thereby reducing localized concentration polarization. In addition, the Li-ion diffusion coefficient (*D*_Li^+^_) is determined by galvanostatic intermittent titration technique (GITT). For QSPE-AL, the conformal F-rich CEI and the tight electrode–electrolyte contact enabled by *in situ* polymerization reduce the energy barrier for Li^+^ migration (Fig. S8). As cycling progresses, the high-response regions are primarily concentrated on the outer layer of the particles and exhibit a continuous, smooth gradient distribution, rather than the distinct localized concentration observed in QSPE. This suggests that interfacial stress is uniformly released across the entire particle surface, rather than being concentrated at local grain boundaries. With deep cycling, compared to the distinct localized high-stress hotspots observed in QSPE, the stress distribution with transition metal in the particles in QSPE-AL is smoother and more uniform overall, with only a limited increase in response in the outer layer. This indicates a significant improvement in the mechanical stability of the particles and further demonstrates the stress-buffering effect of the LiF-enriched CEI. For QSPE-AL, the optimized interface can gradually relieve changes in mechanical stress on the material during long-term cycling, thereby reducing the formation of microcracks, minimizing side reactions, and forming a stable interface. As a result, batteries undergoing long-cycle testing in QSPE-AL have a significantly lower impedance increase than batteries tested according to cycle testing in QSPE (Fig. 4c). This is more clearly demonstrated in the distribution of relaxation time (DRT), where cells cycled in QSPE-AL have weaker contact impedance, interfacial impedance, and charge transfer (S3 region) impedance than QSPE (Fig. 4d), indicating that ionic clusters derived with more F-rich components can enhance interfacial stability and cycling stability.

## Conclusions

In this study, by regulating the localized solvation structure in a quasi-solid-state electrolyte, we successfully developed an anion-dominated localized high-concentration electrolyte system (QSPE-AL), which enabled the formation of a LiF-rich cathode electrolyte interphase (CEI) layer on the surface of high-nickel cathodes. Raman spectroscopy results indicate that the anion coordination is enhanced in QSPE-AL, while the free solvent content is markedly reduced, which may promote the preferential participation of anions in interfacial reactions. XPS and EPMA analyses further confirm that QSPE-AL promotes the formation of a more uniform and denser LiF-rich inorganic CEI on the cathode surface. Compared to traditional organic-dominated CEIs, the LiF-rich CEI exhibits higher mechanical strength and interfacial stability, effectively buffering the volumetric strain of high-nickel cathodes during cycling, suppressing continuous electrolyte penetration along grain boundaries, and mitigating the release of lattice oxygen and interfacial side reactions. COMSOL simulation results further reveal that the LiF-rich CEI can uniformly disperse local stresses within the particles, reducing concentration polarization and stress concentration, thereby effectively suppressing the initiation and propagation of microcracks in the cathode particles. Benefiting from the stable, continuous ion transport channels and stable interfaces formed by the *in situ* polymerized polymers and inorganic particles, the NCM622‖Li battery using the QSPE-AL electrolyte exhibited excellent cycling stability, maintaining a capacity retention of ∼88% after 300 cycles and achieving stable cycling for over 1000 cycles. This work reveals an interfacial stabilization mechanism in which an anion-dominated solvation structure forms a LiF-rich CEI, thereby homogenizing cathode stress to suppress microcracking. It provides new insights for the interface design and longevity enhancement of high-voltage quasi-solid-state lithium–metal batteries.

## Author contributions

Z. B. Z. designed the experiments and wrote the paper. Z. B. Z. designed and drew the figures. Z. B. Z. and X. F. L. performed the experiments. Z. B. Z. and X. F. L. supervised the project. Y. Z. Z. and J. W. discussed the results and commented on the manuscript.

## Conflicts of interest

There are no conflicts to declare.

## Supplementary Material

SC-OLF-D6SC04863C-s001

## Data Availability

The data underlying this study are available in the published article and its supplementary information (SI). Supplementary information: detailed experimental procedures for cell fabrication, electrochemical measurements, together with additional data on battery performance, composition, and structural characterization. See DOI: https://doi.org/10.1039/d6sc04863c.
