# Supplementary material for: Anion-regulated solvation chemistry constructs LiF-rich CEI for suppressing cathode microcracking in lithium–metal batteries
Source: Chem Sci. 2026 Jul 16;17(35):16882–91. doi: 10.1039/d6sc04863c (PMC13386865; doi:10.1039/d6sc04863c)
Supplement: Supplementary file 1 [file SC-017-D6SC04863C-s001.pdf]

Supplementary Information (SI) for Chemical Science.
This journal is © The Royal Society of Chemistry 2026

Supplementary information

Anion-regulated solvation chemistry constructs LiF-Rich CEI for suppressing cathode microcracking in Lithium-Metal Batteries

Zibo Zhang, [a,*] Xiaofei Liu, [d] Yongzheng Zhang, [e] and Jian Wang [b,c,*]

[a] School of Chemical Engineering, University of Science and Technology Liaoning, Anshan, Liaoning 114051, PR China

E-mail: zhangzb@ustl.edu.cn

[b] Helmholtz Institute Ulm (HIU) D89081 Ulm, Germany

[c] Karlsruhe Institute of Technology (KIT) D76021 Karlsruhe, Germany

E-mail: jian.wang@kit.edu

[d] Energy-Saving Building Materials Collaborative Innovation Center of Henan Province, Xinyang Normal University, Xinyang, P. R. China

[e] School of Textile and Clothing, Nantong University, Nantong 226019, China

Experiments

Preparation of QSPE and QSPE-AL

The polymer electrolyte is prepared based on our previous method.[1] 1,1,2,2-Tetrafluoroethyl-2,2,3,3-tetrafluoropropylether (TTE, DoDoChem, 99.8%), Fumed aluminum oxide (Al_2O_3, AEROXIDE Alu C), Lithium bis(fluorosulfonyl)imide (LiFSI, DoDoChem, 99.9%), 1,2-dimethoxy-ethan (DME, DoDoChem, 99.9%), AIBN (Aladdin, 98%), Poly(ethylene glycol) diacrylate monomers (PEGDA, Aladdin, Mw=400 g mol^{-1}). The LHCE electrolyte was first prepared at the ratio of (LIFSI: 1.2TTE: 3DME).[2] 5wt% fumed aluminum oxide was added to LHCE to create a stable

suspension electrolyte (QSPE-AL). To create a precursor solution of QSPE/QSPE-AL, PEGDA was added to LHCE/LHCE-AL at 6 wt% for polymerization, and 1 wt% AIBN (in relative to the mass of PEGDA) was added. The composite electrolyte membrane was constructed using the cellulose separator as the skeleton, and the preparation of the QSPE membrane was created using aforementioned heating process by dropping the precursor solution onto the cellulose separator skeleton.

Materials Characterization

Scanning electron microscopy (SEM, JEOL JSM-7500FA) was used to analyze the microstructure of the as-prepared QSPE. Fourier transform infrared spectra of the samples were recorded on a Thermo Scientific/Nicolet iS50 spectrometer in the wavenumber range of 500-2000 cm^{-1}. An ESCALAB 250Xi spectrometer with Al Kα excitation (1486.6 eV) was used to perform X-ray photoelectron spectroscopy (XPS) of the cathode and anode both before and after cycling. Electron probe microanalysis (EPMA, JXA 8530F, PLUS) revealed the microstructure and chemical composition of the cross-sectional cathode surface. All electrodes were cleaned with 1,2-dimethoxyethane (DME, Sigma Aldrich, 99%) to remove electrolyte salts and residual solvents prior to testing.

Computational Details

All DFT calculations were carried out in the Gaussian (G09)[3] software package utilizing Becke's three-parameter hybrid technique with the Lee-Yang-Parr correlation functional (B3LYP).[4] At the 6- 31 G (d) level, the geometrical structures and vibrational modes were determined.

Battery assembly and characterization

For full-cell assembly, the cathode slurry was prepared by mixing commercial single-crystal NCM622, PVDF, and Super P with a mass ratio of 80:10:10. The mixture was stirred continuously for 24 h to obtain a homogeneous slurry, which was then coated onto one side of an aluminum foil current collector and subsequently dried under

vacuum at 80 °C for 8 h. All CR2032 coin cells were assembled in a high-purity argon-filled glove box. Prior to polymerization, the assembled cells were maintained at room temperature for 2 h to ensure sufficient infiltration of the precursor solution into the electrodes, followed by heating at 60 °C for 30 min to complete the polymerization process. The galvanostatic charge–discharge cycling and rate capability of the single-crystal NCM622/QSPE/Li cells were evaluated at 25 °C within a voltage window of 2.8–4.3 V using a multichannel battery testing system (LAND CT-2001A). The finite element simulations were conducted using COMSOL Multiohysics.[5]

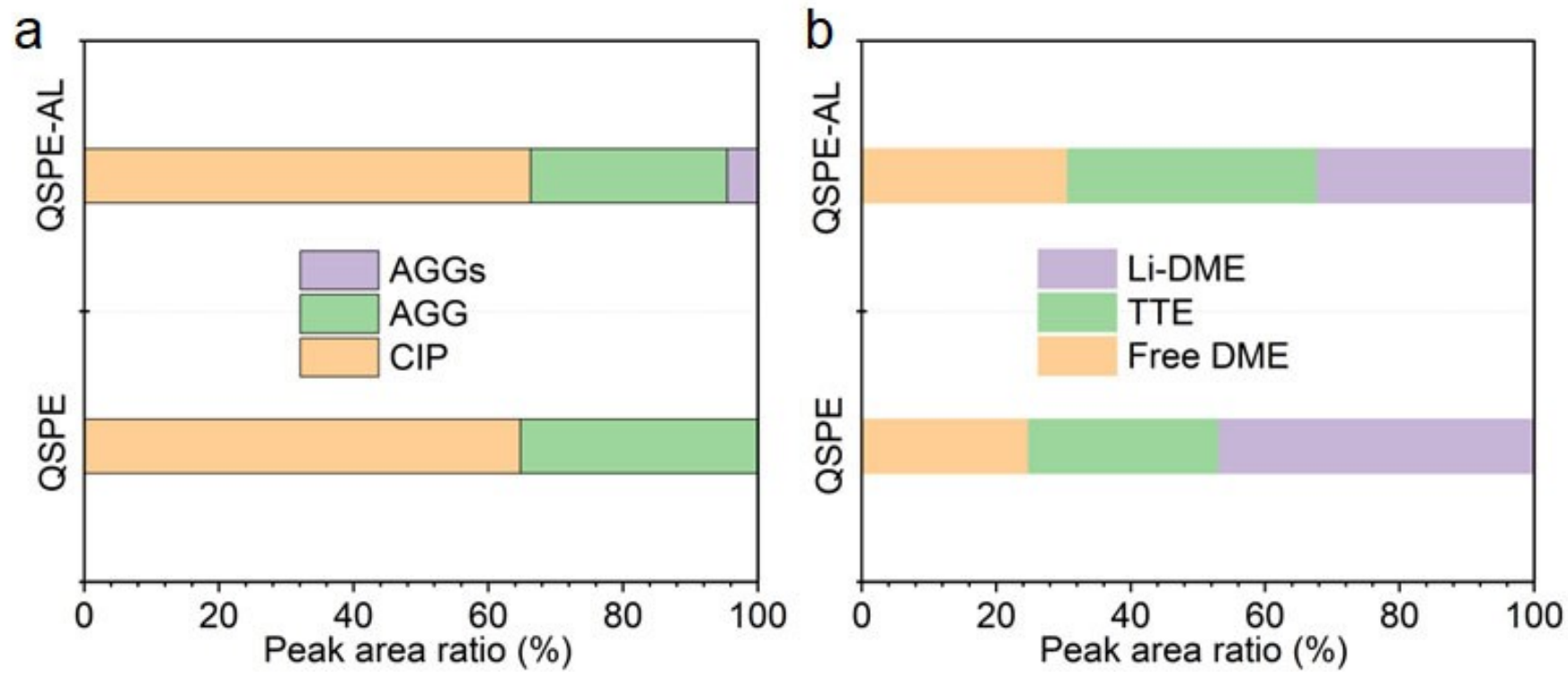

Figure S1. (a-b) Statistical results on the proportions of different solvation structures based on the integration of Raman peak areas.

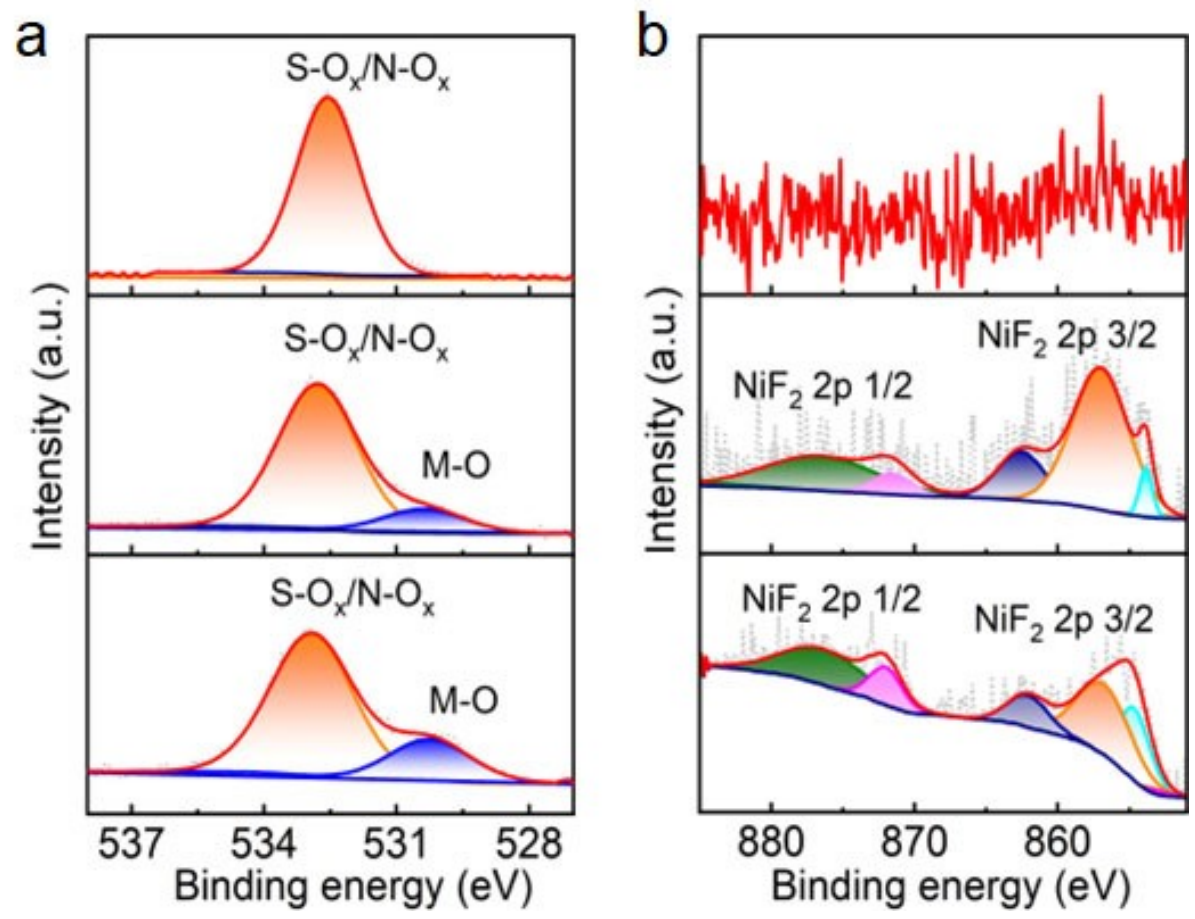

Figure S2. O 1s and Ni 2p XPS spectra of NCM622 before and after etching with QSPE.

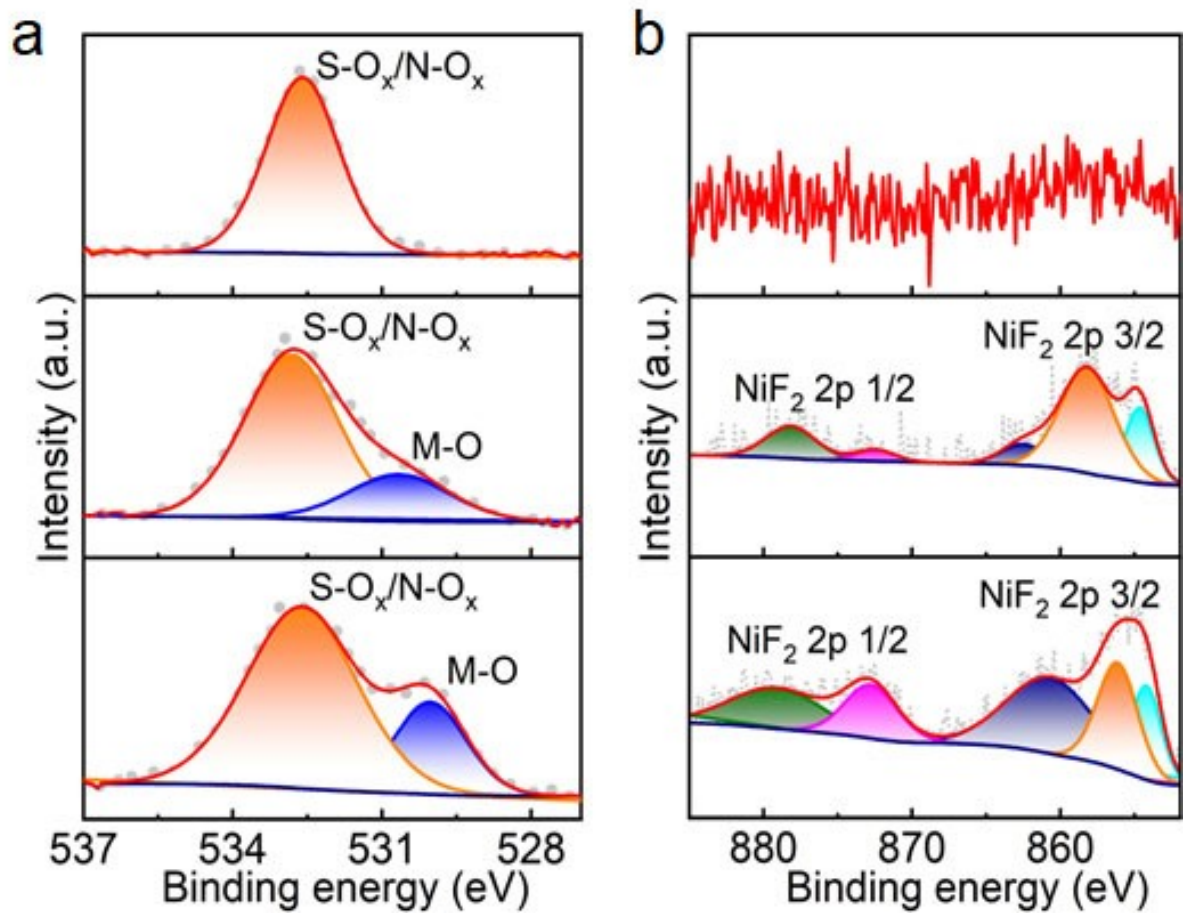

Figure S3. O 1s and Ni 2p XPS spectra of NCM622 before and after etching with QSPE-AL.

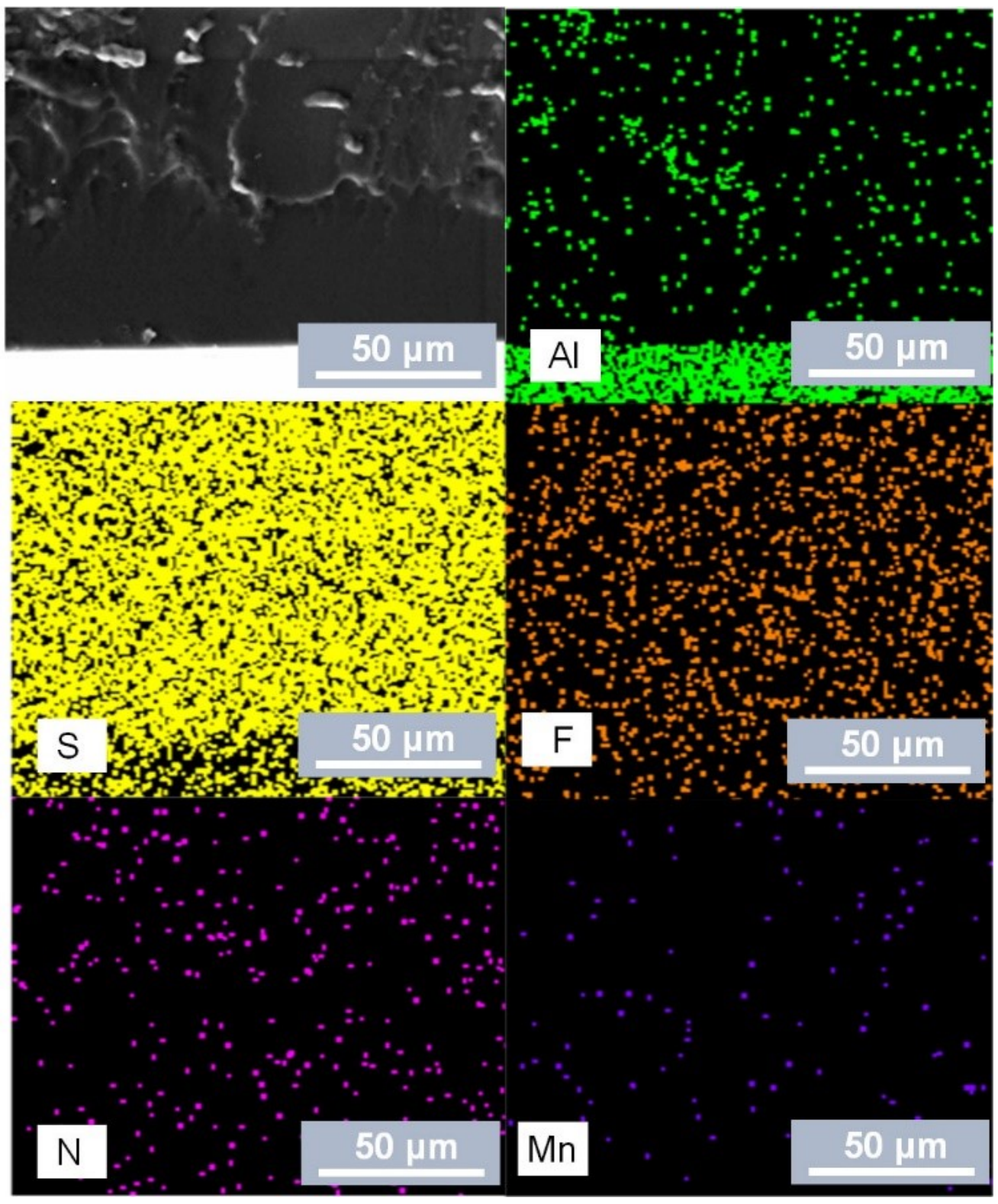

Figure S4. Cross-section SEM image of cathode/QSPE-AL interface and corresponding EDS mapping.

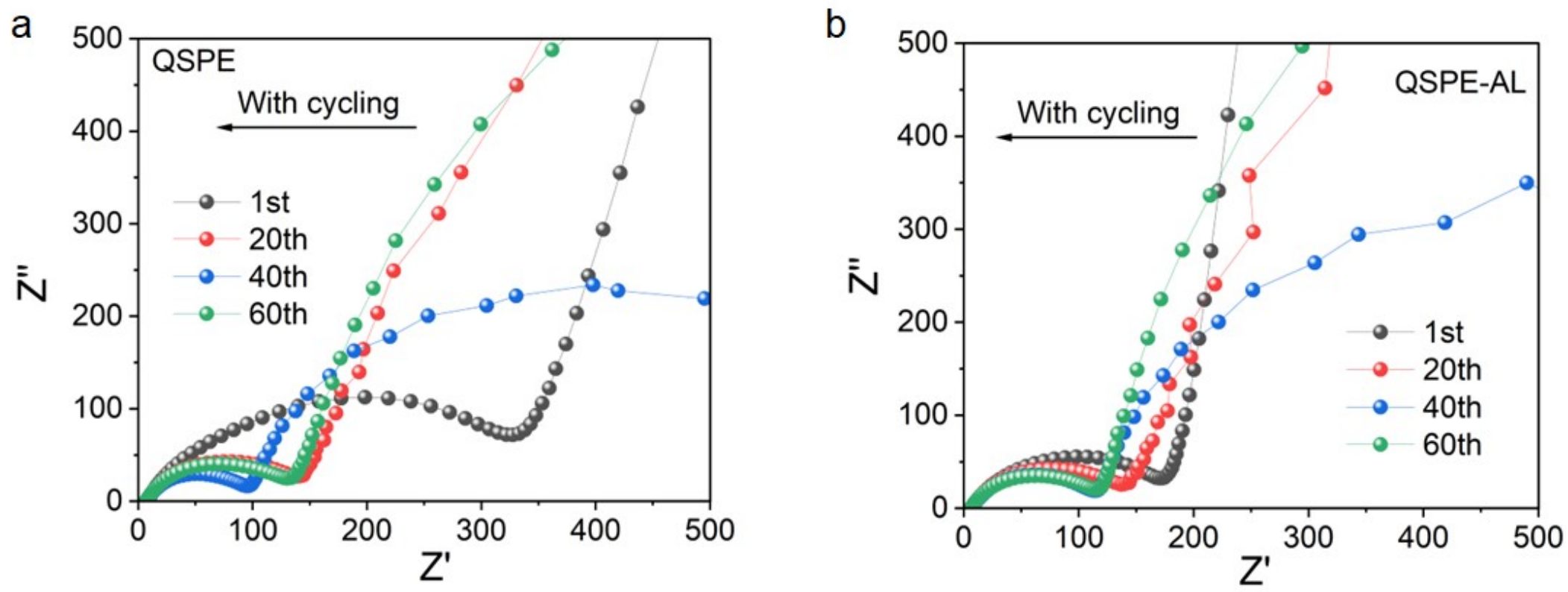

Figure S5. EIS spectra of Li||NCM622 cell in different cycles with different electrolytes.

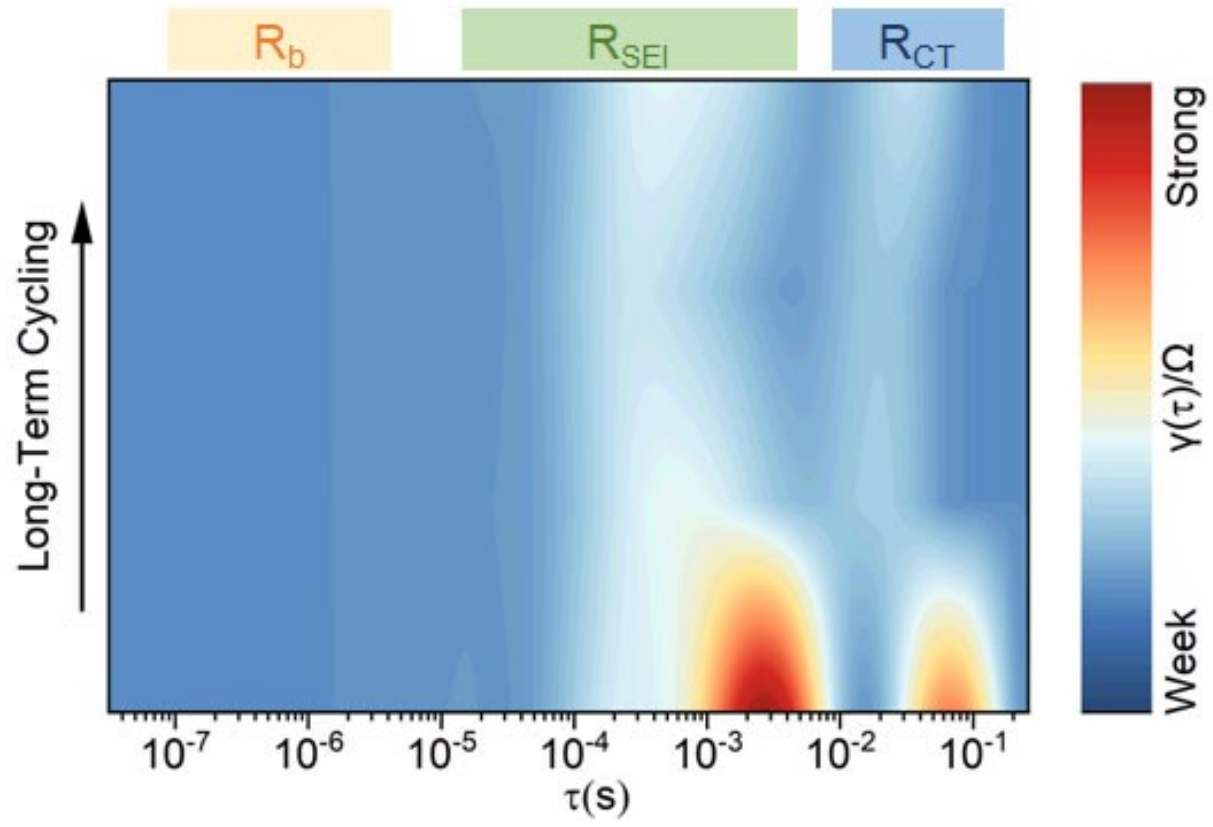

Figure S6. The distribution of relaxation time (DRT) analysis of EIS Nyquist plots for different cycles with QSPE.

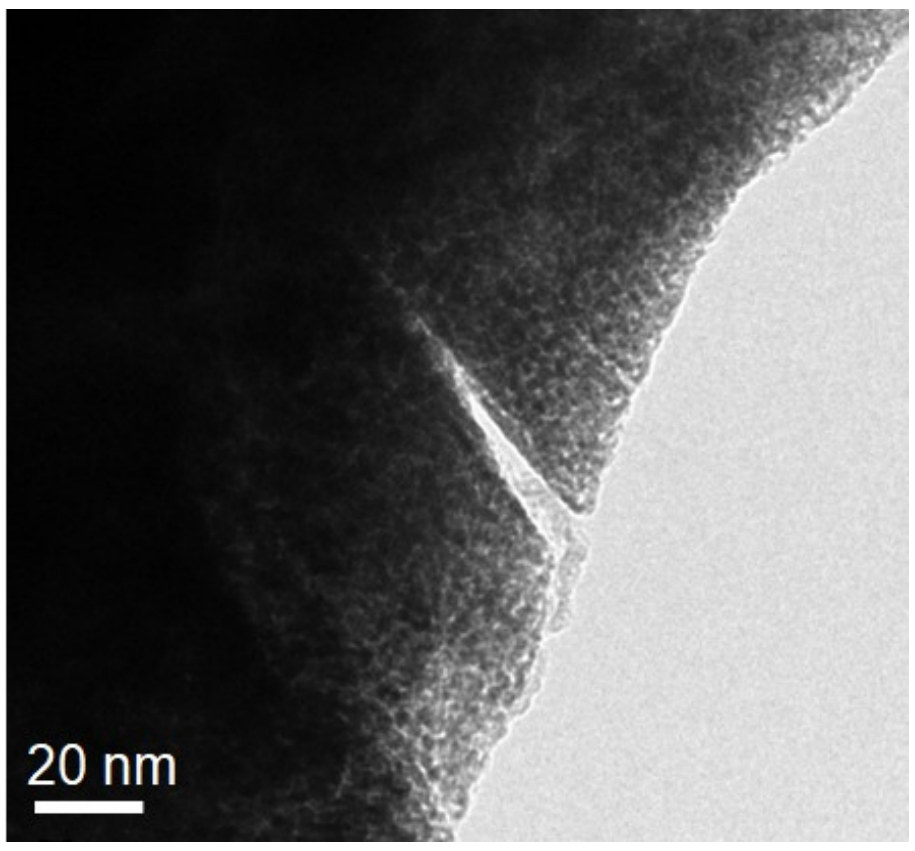

Figure S7. TEM images of NCM622 after 50 cycles with QSPE.

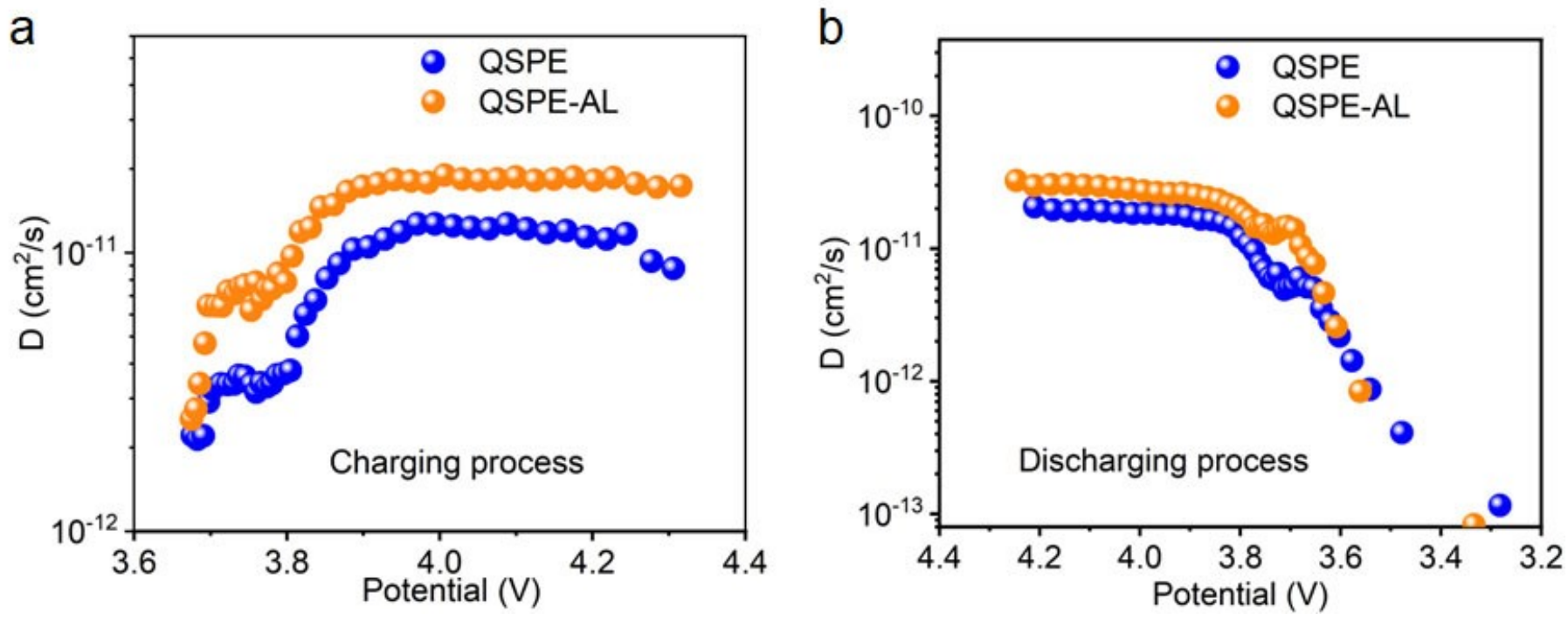

Figure S8. Li chemical diffusion coefficients as a function of voltage from GITT with charge (a) and discharge (b) state.

References

1 Z. Zhang, X. Liu, D. Wang, H. Qin, X. He, B. Gao, G. Li, W. Zheng, Z. Zhuang, G. Yang and X. Ou, Energy Storage Materials, 2024, 69.

2 X. Ren, L. Zou, X. Cao, M. H. Engelhard, W. Liu, S. D. Burton, H. Lee, C. Niu, B. E. Matthews, Z. Zhu, C. Wang, B. W. Arey, J. Xiao, J. Liu, J.-G. Zhang and W. Xu, Joule, 2019, 3, 1662-1676.

3 M. J. Frisch, G. W. Trucks, H. B. Schlegel, G. E. Scuseria, M. A. Robb, J. R. Cheeseman, G. Scalmani, V. Barone, B. Mennucci and G. A. Petersson, 2010.

4 A. D. Becke, The Journal of Chemical Physics, 1993, 98, 5648-5652.

5 W. Ai, B. Wu and E. Martínez-Pañeda, J. Power Sources, 2022, 544.
